# Rheology in the Presence of Carbon Dioxide (CO_2_) to Study the Melt Behavior of Chemically Modified Polylactide (PLA)

**DOI:** 10.3390/polym12051108

**Published:** 2020-05-13

**Authors:** Dominik Dörr, Tobias Standau, Svenja Murillo Castellón, Christian Bonten, Volker Altstädt

**Affiliations:** 1 Department of Polymer Engineering, University of Bayreuth, Universitätsstraße 30, 95447 Bayreuth, Germany; dominik.doerr@uni-bayreuth.de (D.D.); tobias.standau@uni-bayreuth.de (T.S.); 2 Institut für Kunststofftechnik, University of Stuttgart, Pfaffenwaldring 32, 70569 Stuttgart, Germany; svenja.murillo.castellon@ikt.uni-stuttgart.de (S.M.C.); christian.bonten@ikt.uni-stuttgart.de (C.B.); 3 Bavarian Polymer Institute, Universitätsstraße 30, 95447 Bayreuth, Germany

**Keywords:** rheology, chain extender, polylactide acid, polylactide, carbon dioxide, chemical modification, pressure cell, peroxides

## Abstract

For the preparation of polylactide (PLA)-based foams, it is commonly necessary to increase the melt strength of the polymer. Additives such as chain extenders (CE) or peroxides are often used to build up the molecular weight by branching or even crosslinking during reactive extrusion. Furthermore, a blowing agent with a low molecular weight, such as carbon dioxide (CO_2_), is introduced in the foaming process, which might affect the reactivity during extrusion. Offline rheological tests can help to measure and better understand the kinetics of the reaction, especially the reaction between the polymer and the chemical modifier. However, rheological measurements are mostly done in an inert nitrogen atmosphere without an equivalent gas loading of the polymer melt, like during the corresponding reactive extrusion process. Therefore, the influence of the blowing agent itself is not considered within these standard rheological measurements. Thus, in this study, a rheometer equipped with a pressure cell is used to conduct rheological measurements of neat and chemical-modified polymers in the presence of CO_2_ at pressures up to 40 bar. The specific effects of CO_2_ at elevated pressure on the reactivity between the polymer and the chemical modifiers (an organic peroxide and as second choice, an epoxy-based CE) were investigated and compared. It could be shown in the rheological experiments that the reactivity of the chain extender is reduced in the presence of CO_2_, while the peroxide is less affected. Finally, it was possible to detect the recrystallization temperature T_rc_ of the unmodified and unbranched sample by the torque maximum in the rheometer, representing the tear off of the stamp from the sample. T_rc_ was about 13 K lower in the CO_2_-loaded sample. Furthermore, it was possible to detect the influences of branching and gas loading simultaneously. Here the influence of the branching on T_rc_ was much higher in comparison to a gas loading.

## 1. Introduction

Polylactide (PLA) is often referred as one of the most promising biopolymers as it possesses similar properties to polystyrene, but has a lower carbon footprint [1,2]. However, the lack of sufficient melt strength is often named as an issue and therefore it is often chemically modified in reactive extrusion processes [3]. Regarding chemical modification, it can be distinguished between two approaches, which are (i) a functional group reaction and, (ii) a free radical reaction. The first can be achieved by the addition of epoxides, oxazolines, anhydrides or isocyanates, while the latter can be induced by the use of peroxides. By the use of these modifiers, chain extension, branching and/or crosslinking can be expected, depending on functionality and concentration [4,5,6].

**Figure 1:**
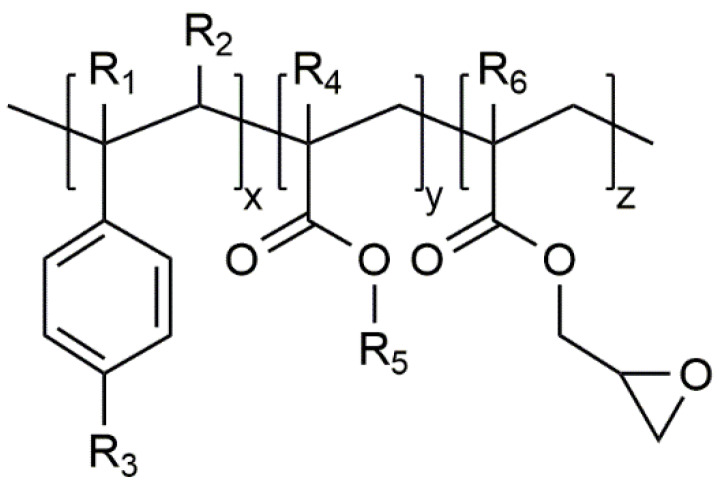
General structure of the styrene–acrylic multi-functional chain extenders according [7].

The recycling industry uses chemical modification during reactive extrusion to counteract chain degradation at high temperatures and the resulting change of properties [7]. To increase the melt strength, modification is often used in the polymer foaming processes in the presence of blowing agents such as CO_2_. Furthermore, in the case of bead foams, the use of a chain extender (CE) can improve or enable their moldability [3,8,9]. Commonly polymeric chain extenders, mostly multi-functional styrene–acrylic-based polymers, are used in the industry, due to their easy dosing and broad processing window [10,11]. The general chemical structure is shown in Figure 1: 

Villalobos et al. [7] showed, that the organic moiety R_1_ to R_5_ are H, CH_3_, a higher alkyl group or combinations of them, R_6_ is an alkyl group and *x*, *y*, and *z* are each between 1 and 20. The number of reactive epoxy groups is higher than four (f_n_ > 4). These epoxy groups react with the polyester groups of the polymer and consequently, long chain branched material can be achieved. By a higher amount of the chain extender, the polymer can even crosslink more easily. 

On the other hand, peroxides usually react much more randomly as they decompose under heat to form the reactive species. Dicumyl peroxide was used before in order to modify PLA. Södergård [12] stated that for concentrations below 0.25 wt.% branching is dominant in PLA, while above this concentration crosslinking happens more likely. 

A change of the polymer topology, caused either by extension, branching or crosslinking, directly influences the rheological behavior of the polymer melt. Besides that, the applied pressure as well as the blowing agent’s diffusion and dissolution process, changes the viscosity of the polymer. An increased pressure leads to an increased viscosity due to the reduction of the free volume [13,14]. This observation leads to the pressure shift factor by combining the free volume equation with the Barus [15] equation [13,16,17]:

(1) $$\alpha_{p}\left(p\right)=\frac{\eta_{0}\left(p\right)}{\eta_{0}\left(p_{0}\right)}=\exp\;\left[\beta \;\left(p-p_{0}\right)\right]$$

with the pressure coefficient β.

In comparison, the blowing agent itself decreases the viscosity [16]. The dissolved gas molecules act as plasticizers and the polymer chains can move more easily. In general, the viscosity of the polymer gas mixture is depending on the amount of dissolved gas molecules [17]. Fujita and Kishimoto [18] deduced a relation between the shift factor and solved gases based on the free volume theory and the Doolittle equation. In combination with the Chow-equation [19], the shift factor of the concentration can be derived:

(2) $$\alpha_{c}\left(c\right)=\exp\frac{\left(\frac{-c}{f}\right)}{\frac{f}{\theta }+c}$$

with the concentration c, fractional free volume f and the contribution of the dissolved gas to the increase of free volume θ. Unlike the other parameters the solved gas affects the vertical shift factor b, due to change of the concentration [17]. This vertical shift factor is defined as:

(3) $$b_{c}\left(c\right)=\frac{c_{pol}\left(c\right)}{p_{pol}\;\left(c_{0}\right)}$$

where $p_{pol}$ is the density of the polymer gas solution and $c_{pol}$ is the polymer concentration of the solution that is defined as $c_{pol}\left(c\right)=\frac{W_{pol}}{V_{m}}$.

Besides viscosity, the dissolved gas influences other aspects of the polymer as well. While increasing the gas content, both the T_g_ as well as the T_m_ is shifted towards lower temperatures [20]. The dissolved gas affects the free volume and therefore the molecular mobility is expected to be higher. Takada et al. [21] investigated the influence of dissolved CO_2_ on the crystallization rate. The overall isothermal crystallization rate is increased, but this depends on the reduction of T_g_ and T_m_. Only if the reduction of T_g_ is higher than the reduction of T_m_ the crystallization rate increases, otherwise it decreases. This result could be transferred to all semi-crystalline polymers [16,21]. Furthermore, the degree of crystallinity is reduced in the presence of CO_2_ and additionally with increasing content of chain extender [22,23,24].

To the authors’ best knowledge, there is no publication about the influence of CO_2_ at elevated pressures on the reactivity of a chemical-modified system and the resulting viscosity. This study should add more information to the publications about the influence of CO_2_ on the viscosity [16,25] and our previous publications about the influence of a chemical modification on the rheological properties [3,26], and should give an insight about the reactivity of different chemical modifications at an elevated CO_2_ pressure. Therefore, PLA is modified with two different commercially available modifiers and analyzed via rotational rheology with and without the presence of high-pressure CO_2_.

## 2. Materials and Methods

This work is based on commercially available materials. The used PLA material is a grade allocated from NatureWorks LLC (Minnetonka, MN, USA), namely Ingeo 4044D with a D-content of about 4%. The polymer was modified with two different chemical modifiers. First, a dicumyl peroxide (DCUP) from Sigma Aldrich (St. Louis, MO, USA) and secondly, a multifunctional epoxy oligomer chain extender (Joncryl® 4468) provided by BASF SE (Ludwigshafen, Germany). The polymer was vacuum dried at 80 °C for 16 h prior use. Both modifiers were added by reactive extrusion with a ZSK 26 twin-screw extruder from Coperion GmbH (Stuttgart, Germany). Temperature was set between 180 and 200 °C at a screw speed of 150 rpm and a throughput of 7 kg/h. The concentrations were selected based on pre-trials with 0.7 wt.% for the chain extender and 0.2 wt.% for the peroxide.

Standard time and frequency sweeps were carried out with plate-plate method on MCR 702 from Anton Paar GmbH (Graz, Austria) on melt pressed samples with a diameter of 25 mm and a gap size of 1.0 mm under nitrogen atmosphere at 180 °C. The time sweeps were carried out at a constant strain of 5% and a constant frequency of 1 rad/s. The frequency sweeps were carried out at a constant strain of 5% and in a frequency range from 1 rad/s to 100 rad/s.

Rotational tests under carbon dioxide atmosphere were performed on a MCR 301 rheometer from Anton Paar GmbH (Graz, Austria) equipped with a high-pressure cell on melt-pressed samples with a diameter of 25 mm and a gap size of 0.8 mm at 180 °C. Here, three different steps were conducted on each material with an atmospheric pressure and 40 bar, respectively. First the diffusion of carbon dioxide was measured every 5 min for 30 s at a constant shear of 1 s^−1^. The first measurement point was extended to 1 min, whereby the pressure was applied after 30 s. In the second step, 1 min stressing tests with a shear rate of 0.3 s^−1^, 1 s^−1^, 3 s^−1^, 5 s^−1^ and 10 s^−1^ with a break of 0.5 min between each measurement. Finally, the sample was cooled down with a constant cooling rate of 1 K/min and a constant shear rate of 1 s^−1^ to investigate the recrystallization behavior under gas loading.

The rheological samples used for the measurements were fabricated with *a hydraulic press PW10*, made by Paul-Otto Weber GmbH (Remshalden, Germany). The polymer pellets were filled into a mold, which was exposed to a temperature of 200 °C. After 2 min, a pressing force of 50 kN was applied for 2 min. Afterwards, the mold was fast cooled in a water-cooled press with an initial pressing force of 50 kN. The prepared samples were stored in a desiccator and measured within the next 24 h.

HFIP-GPC measurement was performed on an instrument having 3 PSS-PFG gel columns (particle size = 7 µm) with porosity range from 100 to 300 Å (PSS GmbH, Mainz, Germany) coupled with a refractive index detector (Gynkotek LTD, Bristow, VA, USA). HFIP (HPLC grade, with 4.8 g potassium trifluoroacetate in 600 mL HFIP) was used as dissolving and eluting solvent with a flow rate of 0.5 mL/min. An internal standard toluene (HPLC grade) was used. The calibration was done with narrowly distributed poly (methyl methacrylate) (PMMA) homopolymers (PSS calibration kit). The injection volume was 20 µL and the measurement was conducted at room temperature. The polydispersity index (PDI) was automatically calculated as quotient from ass average molar mass (M_w_) and umber average molar mass (M_n_) after the measurement. The deviations between the measurements were less than 5%. 

## 3. Results and Discussion

**Figure 2:**
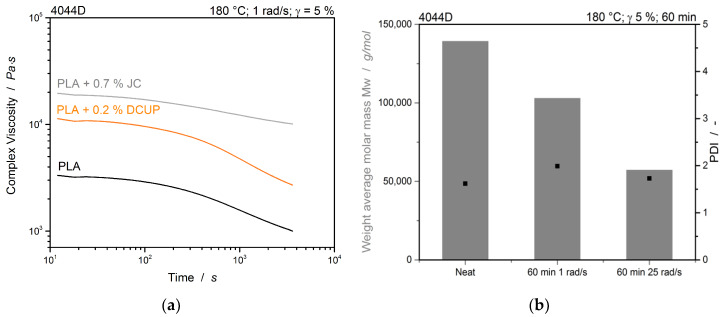
(**a**) Time sweeps and (**b**) The weight average molar mass M_w_ and polydispersity (PDI) before and after the time sweep tested with different frequencies.

The rheological properties of the different PLA are investigated, with and without peroxide and chain extender. First, the different standard oscillatory tests are conducted to get a general rheological characterization of the different polymers. The linear viscoelastic range was investigated with an amplitude sweep at a constant temperature of 180 °C and a constant frequency of 1 rad/s. For the following trials, the strain was set to 5%. The investigation of the polymer time sweeps were conducted at constant temperature, strain and frequency. The result of the time sweep is shown in Figure 2a.

Under shear, the unmodified polymer presents a time stability for only few minutes. Afterwards, the polymer starts to degrade, which is typically for polyesters like PLA. The decrease in molecular weight of the unmodified sample is measured via GPC before and after the time sweep. To verify the influence of the shear, another time sweep is performed at a frequency of 25 rad/s. The weight average molar mass M_w_ and the polydispersity are shown in Figure 2b. 

It is obvious that during the measurement, the molecular weight decreases by about 25% from 139,320 g/mol to 103,030 g/mol. This decrease increases with a higher frequency of 25 rad/s to approx. 60% and a molecular weight of only 57,349 g/mol. The polydispersity is nearly constant between 1.6 and 1.9, showing an overall degradation of the polymer. In our previous study, we could show that in the absence of shear but under applied pressure (CO_2_), no reaction occurred over a long period of several hours during isothermal saturation of PLA with CO_2_ in an autoclave [27]. However, shear-induced degradation can be expected in the modified PLA samples, but due to the nonlinear character, liable GPC measurements were not possible. Here, it can be assumed that the polymer modified with chain extender still contains reactive species. For the CE-modified PLA, it is obvious that the viscosity decreases to a much lesser extent. Hence, it is less affected by the shear induced degradation and the molecular weight is likely to remain stable to a certain extent. A simultaneous thermal degradation and chain extension takes place on the chain extender-modified material, whereby the peroxide material seems to be already reacted completely during the modification and degradation with a similar extent like the unmodified PLA occurs during the measurement (cf. Figure 2a). This can also be attributed to the specially used grade, which has been specially developed by the manufacturer for use with Joncryl^®^ [28]. 

**Figure 3:**
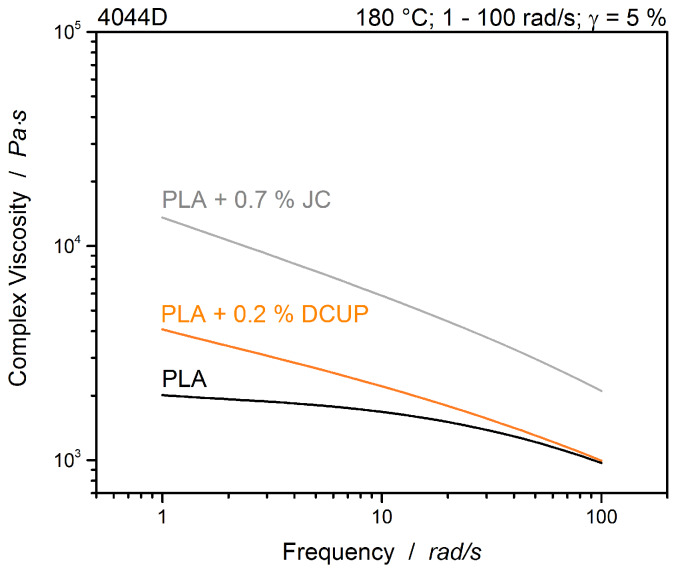
Frequency sweeps of modified and unmodified PLA.

The results of oscillatory frequency sweeps at 180 °C are shown in Figure 3. At low frequencies, the complex viscosity is higher than the neat PLA and it can be assumed, that both modified PLAs have a significant higher molecular weight than the neat one. The different flow behaviors can be attributed to different chain topologies. The topological changes of the polymer chains were described in previous studies, as well as the effect on the rheology [26,29,30]. The new results are consistent with the previous studies. 

Rotational measurements with the pressure cell are highly complex. Different influences have to be considered in the evaluation, like the swelling of the sample, delayed rotation transfer through a magnetic coupling and ball-bearing effects of the rheometer (due to special design of the pressure cell, see [25]) as well as the preparation and conduction of the measurement. Therefore, the relative viscosity (relative to the initial viscosity during the diffusion measurement) is plotted and evaluated.

**Figure 4:**
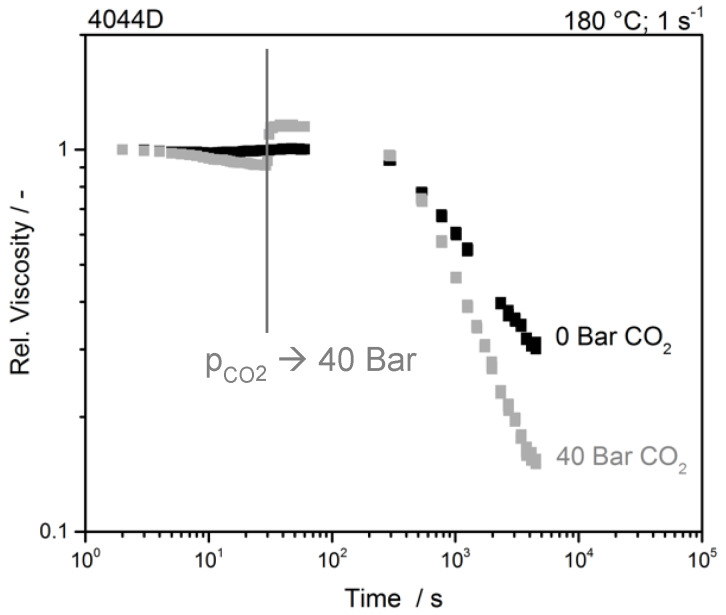
Time sweep of unmodified PLA with and without the presence of CO_2_.

The properties of the gas loaded samples were measured at a constant shear rate with a CO_2_ pressure of 0 bar (1 bar atm) and 40 bar. The results of the unmodified PLA are shown in Figure 4.

The viscosity of the unmodified sample, tested at 0 bar carbon dioxide, is nearly constant within the first minute and then decreases. The viscosity increase of about 30% after applying high pressure of 40 bar (CO_2_) can be explained with the Barus equation (cf. Equation (1)) [15,16]. The pressure dependency β correlates with the difference of glass transition and melt temperature [16]. Subsequently, the pressure sensitivity and increase in viscosity are comparatively low for the used PLA. Besides, the already mentioned thermal degradation of the material, the diffusion and dissolution of the blowing agent into the sample leads to a plasticization and hence a decrease of viscosity. After about 60 min, the measurement is finished, and an 85% lower viscosity of the CO_2_ saturated sample can be seen. When deducting the degradation, CO_2_ reduces the viscosity by approx. 40%.

**Figure 5:**
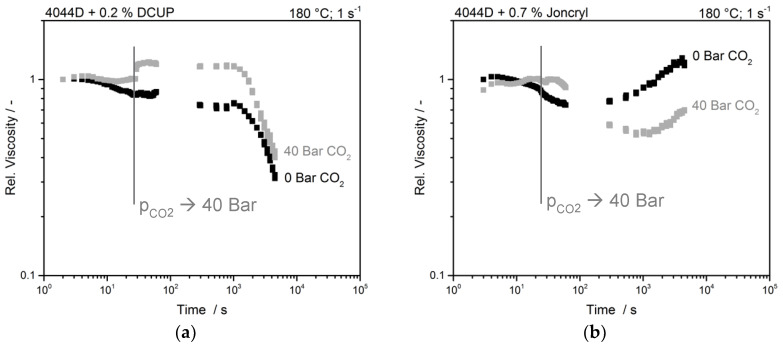
Time sweep of (**a**) peroxide modified and (**b**) chain extender modified PLA with and without the presence of CO_2_.

The processes of diffusion and dissolution of the modified samples are shown in Figure 5.

The peroxide modified sample shows the same behavior after the pressure jump, but in contrast to the unmodified sample the passing time until a steady state is achieved is broadened. With an increased number of branches, the transition time increases as well, but due to the branches, the viscosity effect is less pronounced. The time dependency of branched polymers was first time published by Raps [16] and can be transferred to the PLA system as well. In comparison to the unmodified sample, the peroxide-modified PLA shows a longer time stability, but after about 15 min, the degradation starts. 

Interestingly, the chain-extended sample with Joncryl shows no increase in viscosity after applying the pressure (~30 s) and therefore no pressure dependence. Here, the already strongly developed branched topology could prevent the free volume from decreasing. The general increase of viscosity during the measurement in the sample without CO_2_ states that the reaction of the chain extender still takes place (cf. Figure 2a). The diffusion and dissolution process of CO_2_ and the plasticization of the melt is faster than the branching reaction of the chain extender. Within the first two measurement time frames, the viscosity drops highly and afterwards the viscosity increases. It is possible that the dissolved CO_2_ hinders the overall reaction of the chain extender, hence by a low free volume of the sample, low chain movement is possible. Only after the CO_2_ diffused into the sample, the chain movement increases, and a reaction can take place. However, it seems, that the slope of viscosity increase is flattened, and the overall reaction is slower. Kazarin et al. [31] showed, that the interaction between groups with electron-donating groups (here carbonyl groups) is pronounced. Shi et al. [32] transferred this observation to PLA and the esther group. They assumed that this is the reason for the uptake of over 20 wt.% CO_2_. A high affinity between the reactive groups of the PLA and the blowing agent could reduce the reactivity with the chain extender and slow the reactivity.

**Figure 6:**
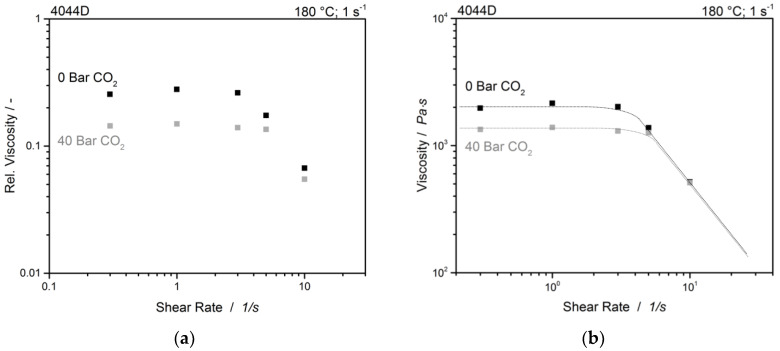
(**a**) Relative viscosities and (**b**) Steady state viscosities at different CO_2_ pressures of unmodified PLA.

The decrease of viscosity by CO_2_ is obvious and the zero-shear values are significantly different from each other in the unmodified sample. The data obtained are consistent with the already shown results. Therefore, only the unmodified graphs are shown in Figure 6. The viscosity is decreased by the presence of CO_2_ by about 40%. The occurrence of a viscosity drop at lower shear rates could be a result of a too-short measuring point and would not occur with a longer measuring time [33].

**Figure 7:**
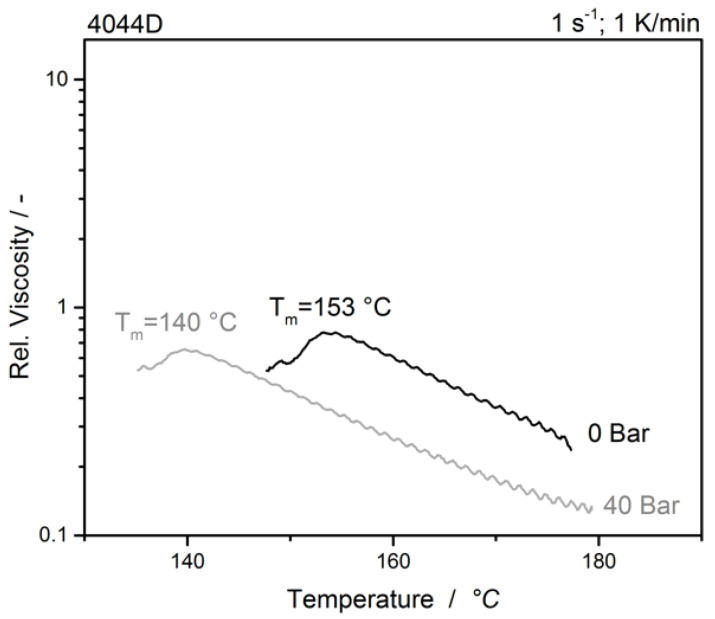
Crystallization of the unmodified sample at two different pressures.

Finally, the effect of dissolved CO_2_ on the crystallization under gas loading was investigated. The fully saturated samples were cooled down with a constant cooling rate of 1 K/min and a constant shear of 1 s^−1^. For the determination of the crystallization temperature, the torque maximum, representing the tear off of the stamp from the sample was chosen. Figure 7 shows the crystallization temperature of the unmodified sample. The recrystallization temperature T_rc_ of about 153 °C fits very well with the typical material properties stated in the technical data sheet [28].

T_rc_ of the saturated sample is reduced by about 13 K compared to the unsaturated sample. This decrease in T_rc_ could be caused by the increased free volume and following a higher of chain mobility [16,23,34].

**Figure 8:**
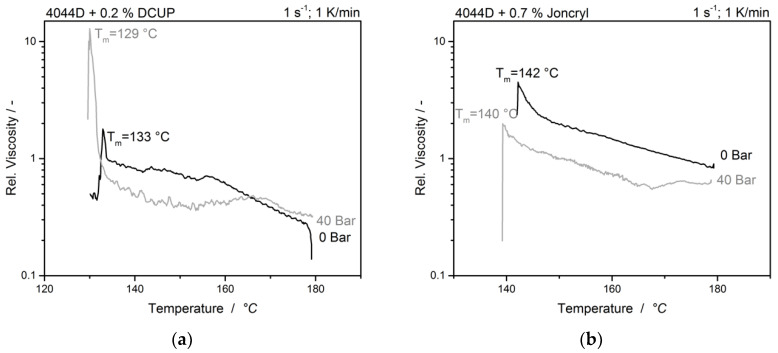
Crystallization of the (**a**) DCUP and (**b**) Joncryl modified samples at two different pressures.

The decrease of T_rc_ by the chemical modified samples without carbon dioxide loading is already shifted to lower temperatures as shown in Figure 8.

Here it can be assumed that the branches hinder the crystallization and packing to crystals takes much more time. This result is contradictory to investigations made by Nofar et al. [23,35], where a nucleating effect of the branches was observed, resulting in a faster recrystallization, but as shown in these publications, the used PLA was a different grade with different recrystallization kinetics. In addition, this effect could also depend on the chain length, with short chains having a nucleating effect, whereas long chains, as in this case, prevent recrystallization as they introduce sterical hindrances. The effect of carbon dioxide on the recrystallization temperature T_rc_ in the branched samples is much less pronounced than in the unbranched sample, which is only a 4 K and 2 K difference, respectively. It can be presumed that the overall recrystallization is already highly reduced by the branches and the recrystallization is generally quite challenging at this branching state.

## 4. Conclusions

To better understand the reactive foam extrusion process, rheological investigations in the presence of carbon dioxide were performed. For these experiments, a special pressure cell was used in order to apply a high CO_2_ pressure during the measurement. An epoxy-based chain extender (CE, Joncryl®) and a peroxide were selected to chemically modify PLA in the rheometer. As expected, the viscosity was decreased by the presence of CO_2_ in all samples. The neat polymer in the rheometer showed both: the dissolution and diffusion of the CO_2_ and the thermal degradation of PLA. In the case of the peroxide modified sample, the degradation is much less pronounced in the presence of CO_2_. The CE modified sample was still reactive during the experiment and it was shown, that the CO_2_ inhibits the reactivity of the CE and the gas diffusion is much faster. These findings are very interesting for reactive foam extrusion, because either a higher amount of CE or a longer retention time is needed to get a branched sample that is comparable to an unfoamed sample. By taking into account, that during reactive foam extrusion higher shear rates and a higher (CO_2_) pressures occur, the shown effects could be more pronounced. Finally, the influence of branches and CO_2_ on the recrystallization was investigated by cooling down the sample and measuring the torque maximum in the rheometer. The CO_2_ decreases the recrystallisation temperature T_rc_ of the neat PLA about 13 K down to 140 °C. Additionally, branches show a very similar decrease in the reduction of T_rc_. Here, the branches hinder the recrystallization of the sample and the influence of CO_2_ on recrystallization is less pronounced. During reactive foam extrusion, a longer retention time could lead to a more branched polymer, resulting in a polymer melt that can be further cooled down.
